# Primordial germ cells as a potential model for understanding (Nutri) epigenetic - metabolic interactions: a mini review

**DOI:** 10.3389/fcell.2025.1576768

**Published:** 2025-04-14

**Authors:** Mariam Ibrahim, Ewa Grochowska, Katarzyna Stadnicka

**Affiliations:** ^1^ Health Sciences Faculty, Ludwik Rydygier Collegium Medicum in Bydgoszcz, Nicolaus Copernicus University in Torun, Bydgoszcz, Poland; ^2^ PBS Doctoral School, Bydgoszcz University of Science and Technology, Bydgoszcz, Poland

**Keywords:** nutritional programming, nutriepigenetic, metabolic processes, PGCs, transgenerational effects

## Abstract

Primordial germ cells (PGCs) are the progenitors of gametes (sperm and eggs), making them crucial for understanding germline transmission and epigenetic modifications, which are critical for studying transgenerational effects of nutrition and metabolic diseases. This is particularly relevant given the growing evidence that environmental factors, such as diet, can influence metabolic disease risk across generations through modulating epigenetic mechanisms, as seen in both human and animal studies. The unique biological and experimental attributes make PGCs in the chicken embryo a potential model for exploring the complex interactions between nutrition, epigenetic inheritance, and metabolic diseases, providing insights that are translatable to metabolic health and disease prevention tactics. This brief review emphasizes the potential of chicken PGCs as a model system to investigate the mechanisms underlying transgenerational metabolic programming.

## 1 Introduction

Epigenetic regulation during development plays a crucial role in cell fate determination, lineage specification, and the establishment of cellular identity. Metabolic diseases such as obesity, type 2 diabetes, and non-alcoholic fatty liver disease are affected by epigenetic mechanisms including DNA methylation, histone modification, and non-coding RNA expression (Nicoletti et al., 2024; Rivera-Aguirre et al., 2023; Gómez de Cedrón et al., 2023). Nutritional factors such as vitamin B12, folate, and choline act as methyl donors or coenzymes for one-carbon metabolism, and their dietary intake can modulate the epigenetic patterns, impacting the onset and progression of metabolic diseases (Nicoletti et al., 2024; Rivera-Aguirre et al., 2023). Endocrine disruptors like phthalates, bisphenol A, pesticides, polychlorinated biphenyls, and dioxins, as well as nutritional imbalances, can induce epigenetic changes in primordial germ cells (PGCs), potentially resulting in altered phenotypes in later generations (Rizzo et al., 2023; Brehm and Flaws, 2019; Brieño-Enríquez et al., 2016). Studies have shown that exposure to metabolic disruptors during prenatal or early life stages can cause metabolic diseases in future generations, underlining the need to understand the epigenetic memory and molecular determinants of these effects (Feroe et al., 2017). A key challenge in the field is identifying model systems that allow researchers to track how specific environmental factors, such as nutrition, trigger epigenetic modifications and subsequent changes in gene expression patterns. These models must enable the study of both immediate effects and potential transmission across generations. Potent models are essential for developing nutritional programming strategies to produce desired traits and implement efficient preventive measures for metabolic diseases. The chicken embryo model offers unique advantages for studying these interactions, as it allows precise temporal control over environmental exposures without maternal confounding effects. However, debate persists regarding the stability and inheritance of environmentally-induced epigenetic changes. While some studies demonstrate transgenerational effects of nutritional interventions (Wu et al., 2019), others question the molecular mechanisms and evolutionary significance of such inheritance (Verdikt and Allard, 2021). This brief review aims to shed light on the potential of chicken PGCs as a model for studying how prenatal nutritional and environmental factors influence epigenetic inheritance in metabolic disorders, and the mechanisms linking environmental signals to specific epigenetic modifications.

## 2 The chicken model for metabolic processes research

Chickens have been considered a useful model to explore the role of adipokine mediated regulation in metabolic and reproductive diseases, with parallels to metabolic diseases in humans (Mellouk et al., 2018). Key adipokines, including adiponectin, visfatin, and chemerin, demonstrate conserved regulatory functions across both species (Mellouk et al., 2018). Chickens constitutively exhibit hyperglycemia despite having normal levels of hyperactive endogenous insulin, requiring large doses of exogenous insulin to induce hypoglycemia, mirroring the insulin resistance seen in human type 2 diabetes pathology (Mellouk et al., 2018; Haselgrübler et al., 2017). Moreover, chickens have been genetically selected for traits such as fatness, which is associated with phenotypic variations in adiposity and metabolic disorders (Resnyk et al., 2017). Additionally, the metabolic genes in chickens are largely conserved with those in humans, and several quantitative trait loci (QTLs) connected to fatness in chickens include genes that link to human obesity or diabetes susceptibility (Mellouk et al., 2018; Nadaf et al., 2009). The chicken’s metabolic system allows for the insights into nutrient metabolism particularly through hepatic lipogenesis and tissue-specific insulin signaling patterns (Mellouk et al., 2018). In both humans and chickens, the liver is the primary site for *de novo* lipogenesis (90%) (Liu et al., 2018). Furthermore, the post-hatch period in chickens is especially useful for studying metabolic programming, as it involves substantial changes in liver metabolism that are comparable to human metabolic processes (Van Every and Schmidt, 2021). Besides, chickens offer a well-established model for researching human lipid metabolism disorders, including non-alcoholic fatty liver disease (Ayala et al., 2009). The robustness of chicken metabolic pathways is demonstrated by the genome-scale metabolic model iES1300, which demonstrates substantial homology with human carbohydrate metabolism networks (Salehabadi et al., 2022).

## 3 Current limitations in understanding metabolic-epigenetic interactions

Current limitations in metabolic-epigenetic research center on three key challenges. The incomplete knowledge about how specific metabolites induce epigenetic changes, like histone acetylation and methylation, and how these changes in turn control metabolic pathways is one of the main limitations. This bidirectional interaction is key in a variety of biological contexts, encompassing embryonic development, cancer, and chronic diseases, however, it is difficult to characterize due to the complexity of these processes and their heterogeneity between cell types and conditions (Milazzotto et al., 2023; Ge et al., 2022; Gómez de Cedrón et al., 2023). Furthermore, the field is impeded by the limited understanding of how epigenetic changes caused by metabolic alterations can be passed down between generations, as seen in studies of paternal transgenerational inheritance of metabolic diseases (Pepin et al., 2022). The potential for targeted nutritional and lifestyle interventions to modulate epigenetic marks and maintain metabolic homeostasis is promising, yet the precise mechanisms and long-term effects of such interventions have yet to be fully understood (Gómez de Cedrón et al., 2023).

## 4 Nutritional programming in chicken model

Growth and development-related metabolic pathways can be optimized through prenatal dietary stimulation. Maternal nutrition, for example, β-carotene supplementation, can influence embryonic development through the growth hormone-insulin-like growth factor axis, promoting liver development and affecting metabolism-related gene expression (Wang et al., 2024). Contrariwise, prenatal protein undernutrition, induced by albumen removal, has been shown to cause long-term alterations in body weight, reproductive performance, and hepatic metabolism, underscoring the vital role that proper prenatal nutrition plays in metabolic programming (Willems et al., 2015).

Understanding the epigenetic changes driven by nutrients is necessary to gain deeper insight into diet-gene interactions. Nutriepigenetics provides insights into improving poultry health and performance by modulating genes associated with immunity, metabolism, and growth (Hassan et al., 2022). The in ovo feeding (IOF) technique, originally designed for vaccine delivery in broiler hatcheries, has evolved into a cost-effective approach for studying early nutrition in chickens (Das et al., 2021). This method now incorporates a variety of substances, including nutrients such as glucose, amino acids, and vitamins, as well as supplements like probiotics, prebiotics, exogenous enzymes, hormones, vaccines, drugs, and nutraceuticals (Das et al., 2021). Given the critical role of embryonic nutrition in regulating tissue and organ development in later stages, in ovo injections and IOF are recognized as powerful tools for implementing targeted nutritional strategies at early developmental stages, and to investigate the effects of injected chemicals and the epigenetic changes they cause. For instance, the administration of L-leucine in ovo has been found to stimulate lipid metabolism and enhance thermotolerance in male chicks under heat stress, indicating a sex-dependent metabolic response (Han et al., 2018).

Dietary methyl donors such as folate, choline, and B vitamins are crucial for DNA methylation, influencing gene expression and disease risk (Anderson et al., 2012). *In ovo* folic acid supplementation has been reported to improve immune function and growth in broilers by modifying histone methylation in immune gene promoters (Li et al., 2016). Furthermore, feeding-based dietary betaine supplementation has been shown to modulate DNA methylation in response to corticosterone-induced hepatic cholesterol accumulation. Key cholesterol gene expression (*HMGCR*, *CYP7A1*) was normalized by reversing corticosterone-induced methylation changes, highlighting the epigenetic influence of diet (Wu et al., 2024). Paternal folate supplementation in chickens has been shown to affect the growth and metabolic profiles of offspring, with changes in lipid and glucose metabolism linked to alterations in spermatozoal and hepatic miRNAs and lncRNAs (Wu et al., 2019). Guo et al. (2024) found that excessive folic acid intake in male chickens can alter sperm DNA methylation (6 mA and 5 mC), increasing hepatic lipogenesis and lipid accumulation while reducing lipolysis in both roosters and their offspring. This study highlights environment-sensitive regions in the sperm epigenome that respond to dietary factors and transmit an epigenomic map, potentially shaping metabolic health in offspring.

Despite the advantages of embryonic manipulations in avian species, there have been relatively few studies on PGCs concerning the transgenerational inheritance effects of epigenetic stimuli.

## 5 Main metabolic-epigenetic crosstalk in chicken germ cells

PGCs in chicken possess unique epigenomic landscape, which, despite sharing some conserved features with mammals, exhibit distinct epigenetic signatures that reflect their evolutionary and developmental pathways, reviewed in (Woo and Han, 2024). In chickens, PGCs are specified by preformation and are influenced by maternally inherited factors, contrasting with the inductive specification seen in mammals (Kress et al., 2024). Unlike mammalian PGCs, chicken PGCs do not experience genome-wide DNA demethylation or a decrease in histone H3K9me2, which are typical features of extensive epigenetic programming in mammals (Kress et al., 2024). Instead, chicken PGCs maintain high levels of 5mC and exhibit a unique epigenetic signature characterized by high global levels of H3K9me3, particularly in inactive genome regions. This signature is progressively established during migration and remains stable in the gonads, indicating a divergence from the basal state resetting observed in mammals. The processes in chicken PGCs are more about chromatin reconfiguration rather than *bona fide* programming, as seen in mammals (Kress et al., 2024). Additionally, the transcription factor Zeb1 and histone methylation regulate *BMP4* expression, highlighting the interplay between genetic and epigenetic factors in PGC development (Zhou et al., 2021). LncRNAs also contribute significantly to chicken PGC development (Jiang et al., 2021). Furthermore, during mitotic arrest, chicken prospermatogonia undergo unique epigenetic programming, characterized by gradual DNA demethylation and histone acetylation, which differs from the mammalian pattern (Choi et al., 2022). These findings underscore the distinct epigenetic landscape of chicken PGCs, which involves a combination of DNA methylation, histone modifications, and non-coding RNAs, all contributing to the regulation of germ cell development and differentiation (Woo and Han, 2024; Rengaraj et al., 2022).

Metabolic regulation in chicken PGCs involves a complex interplay of pathways and factors that ensure proper development and function. Glycolysis is a critical metabolic pathway, with glucose phosphate isomerase (GPI) being essential for maintaining glycolysis and energy supply in chicken PGCs. Knockdown of GPI significantly reduces the expression of glycolysis-related genes and endogenous glucose levels, underscoring its role in PGC proliferation (Rengaraj et al., 2012). Additionally, the transition from glycolysis to oxidative phosphorylation is a key event in PGC formation, indicating a shift in energy metabolism as these cells develop (Zuo et al., 2023). The *C1EIP* gene, regulated by STAT3 and histone acetylation, promotes PGC formation by interacting with ENO1 and inhibiting the Notch signaling pathway (Jin et al., 2020). The TGF-β and Wnt signaling pathways are also activated during PGC formation *in vitro* and *in vivo*, further emphasizing the metabolic and signaling intricacies involved in PGC regulation (Ding et al., 2024). Autophagy, as indicated by the increased number of autolysosomes, is another metabolic process that is enhanced in PGCs, especially following BMP4 induction (Ding et al., 2024). The piRNA pathway also plays a protective role in PGCs, with piRNA pathway genes such as *CIWI* and *CILI* being crucial for maintaining genomic integrity and preventing DNA double-strand breaks (Rengaraj et al., 2014). These pathways collectively underscore the complex metabolic network that supports the development and function of chicken PGCs, integrating energy metabolism, signaling, and genomic protection mechanisms.

Metabolic pathways are intricately linked to epigenetic changes, as metabolites can influence epigenetic mechanisms, and conversely, epigenetic modifications can regulate metabolic processes (Verdikt and Allard, 2021). This metabolic-epigenetic interplay is crucial during early germ cell development, affecting cell fate determination and potentially playing a role in transgenerational epigenetic inheritance (Verdikt and Allard, 2021).

## 6 Chicken PGCs: a tool for transgenerational studies

Chicken PGCs may offer a window into the epigenetic mechanisms that mediate the transgenerational effects of prenatal nutritional interventions. Growing evidence suggests that dietary influences can significantly impact epigenetic marks in PGCs, which are crucial for transgenerational inheritance. The application of nutritional programming in chickens, unlike in mammals, allows for the isolation of nutritional effects without hormonal interference, providing a clearer understanding of its impacts on growth and metabolism (Willems et al., 2015). The unique accessibility of avian PGCs during early development, due to their migration via blood circulation, provides an opportunity for their collection, which is not as easily achievable in mammalian models (Nakamura et al., 2013). Chicken PGCs can be isolated from embryos at various stages of development, each offering unique advantages for research and application. The isolation of PGCs from embryonic blood is commonly performed at HH stages 14–16, where they are abundant in circulation before migrating to the gonadal regions (Dehdilani et al., 2023). Additionally, chicken PGCs can be isolated from the embryonic gonadal regions at later stages, such as HH 26–28, where they have migrated and begun to settle (Zare et al., 2023). Chicken PGCs are characterized by several molecular markers that are crucial for their identification and study such as *SSEA-1*, *EMA-1*, *SSEA-4*, and *SSEA-3* (Mathan et al., 2023). Pluripotency markers such as *POUV*, *SOX2*, and *NANOG*, along with germ cell markers like *DAZL* and *CVH* markers are consistently expressed across various conditions, including fresh isolation, cryopreservation, and *in vitro* culture, indicating the cells’ stability and resilience (Ibrahim et al., 2024). Chicken PGCs are a model for *in vitro* culture. The chicken is the only vertebrate whose PGCs can be stably cultured *in vitro* for an extended period of time (Ichikawa and Horiuchi, 2023). The ability to culture chicken PGCs *in vitro* has been well-documented, with various studies highlighting their resilience and the maintenance of their germline characteristics during long-term culture and cryopreservation (Kong et al., 2018; Ibrahim et al., 2024). The development of optimal culture systems for chicken PGCs has been a focus of several studies comparing the efficiency of different media dedicated to cell expansion and differentiation (Dehdilani et al., 2023). One of the most efficient systems is the feeder-free culture method developed by for expanding chicken PGCs, applied in the research over the last decade (Whyte et al., 2015). Despite the advancements, challenges remain in establishing standardized culture conditions. A primary issue is the inconsistency in protocols across different laboratories, leading to variations in success rates for cell growth and maintenance. These discrepancies make it difficult to replicate and reproduce results reliably. The derivation, expansion, and long-term culture of PGCs appear to depend on multiple factors, including the quality of materials, embryos and incubation quality, the breed of chickens from which PGCs are derived, and the specific combination of culture components essential for PGC survival (Dehdilani et al., 2023). Successful cultivation of chicken PGCs requires specific growth factors and supplements to maintain their developmental potency, stemness, survival, and proliferation (Dehdilani et al., 2023). The absence of these essential components can impair cell growth and viability. Key growth factors include Fibroblast Growth Factor 2 (FGF2), Activin A, BMP4, Insulin-like Growth Factor 1 (IGF-1) and B27 supplement (Miyahara et al., 2016; Whyte et al., 2015; Barkova et al., 2022; Choi et al., 2010).

Additionally, the short-term interval between generations enables tracking the transgenerational effect of studied dietary factors. Artificial insemination technology and the high reproductive capacity of hens, producing up to 300 eggs annually, allow for the generation of enough offspring broilers to study the potential transgenerational impacts of nutritional interventions (Ibrahim et al., 2025). Chickens provide a unique model due to their ability to minimize maternal confounding effects through direct manipulation of egg content, which is not possible in mammalian models (Morisson et al., 2017). This allows for precise control over the nutritional environment during critical developmental periods, facilitating the study of nutritional programming and its transgenerational effects (Morisson et al., 2017). The success of nutritional interventions heavily depends on the selection of suitable delivery techniques and platforms, a condition fulfilled through the application of in ovo injection in chicken embryos. The use of chickens as a model for nutritional rehabilitation, as demonstrated in studies involving dietary interventions in broilers, further underscores their potential as a translational model for human nutritional studies (Baxter et al., 2018). Chickens have been instrumental in advancing knowledge about the role of specific nutrients, such as omega-3 fatty acids, in early life nutritional programming, which can inform strategies to improve human health and productivity (Cherian, 2013).

**FIGURE 1:**
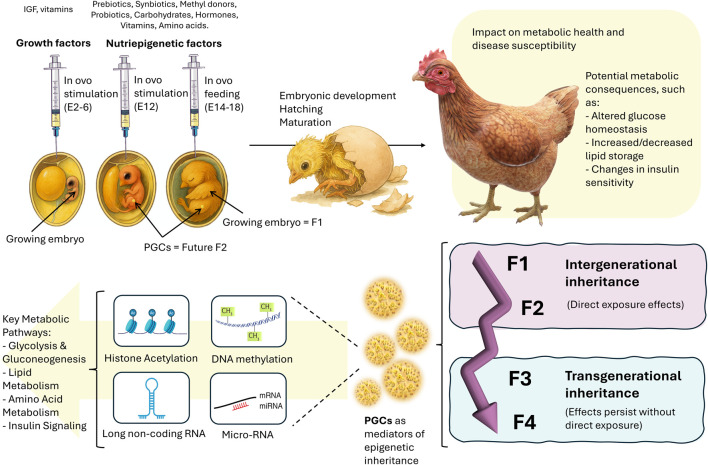
The in ovo model for investigating nutrient-induced metabolic programming in chickens through PGCs. In ovo stimulation (E12) and in ovo feeding (E14-18) introduce nutripigenetic factors (e.g., prebiotics, probiotics, methyl donors, carbohydrates, hormones, vitamins, and amino acids) that influence embryonic development (F1). These interventions can induce epigenetic modifications (including histone acetylation, DNA methylation, long non-coding RNA, and miRNA regulation) that affect key metabolic pathways such as glycolysis, gluconeogenesis, lipid metabolism, amino acid metabolism, and insulin signaling. Changes in metabolic regulation may alter glucose homeostasis, lipid storage, and insulin sensitivity, potentially leading to metabolic disorders. Through primordial germ cells (PGCs), these epigenetic and metabolic effects can be inherited across generations, contributing to intergenerational (F1-F2) and transgenerational (F3-F4) inheritance of metabolic traits. This highlights the potential of chicken PGCs as a valuable model for studying the epigenetic basis of nutrition-induced metabolic diseases.

Recent research by Verdikt et al. has highlighted the interplay between metabolic and epigenetic regulation of PGCs in mammals, particularly in the context of transgenerational epigenetic inheritance (Verdikt and Allard, 2021). Their review suggested that environmental factors may influence epigenetic remodeling in PGCs through metabolic pathways, thereby affecting gene expression. While most studies have focused on mature germ cells, such as sperm and eggs, PGCs remain relatively understudied despite their potential sensitivity to environmental changes. This sensitivity makes PGCs a crucial window for investigating how epigenetic information is transmitted across generations. Another study also hypothesized that the DNA methylome of sperm may show changes in its expression profile in response to high paternal folic acid intake, which has been widely suggested as a methyl donor for the DNA methylation process, and then the altered sperm DNA methylome could transmit certain metabolic and developmental changes from father to offspring (Guo et al., 2024). Although chickens may not serve as an ideal translational model for studying germline programming mechanisms in humans due to species-specific differences, they are highly valuable for investigating multigenerational effects of nutrients, particularly in the context of metabolic processes. The in ovo model allows researchers to explore how nutrients impact epigenetic regulation of metabolic processes, gene expression, and development across generations (Figure 1). This approach provides critical insights into the inheritable effects of key nutrients, which are relevant to human health and the development of other vertebrates.

Overall, the investigation into transgenerational inheritance in chicken PGCs not only enhances our understanding of evolutionary biology and adaptation but also holds potential implications for improving animal breeding and addressing metabolic health issues in broader contexts.

## 7 Conclusion and perspectives

In agreement with Diniz et al. (2024), further advances are essential for translating findings into applications for developmental disorders and understanding the broader implications of early-life nutrition for long-term health outcomes. Therefore, investigation of nutriepigenetic effects transmission through the chicken PGC model has revealed important insights, while also highlighting critical areas for future research: (1) elucidating the molecular mechanisms underlying nutrient-induced epigenetic modifications in PGCs, (2) understanding how these modifications are maintained and transmitted across generations, and (3) determining the conservation of these mechanisms across species. The chicken PGC model offers unique advantages for addressing these questions, particularly through its experimental accessibility and ability to control environmental exposures precisely. It is important to note that this model system should be applied carefully and serve primarily at the very early stages of preclinical trials, providing an initial overview of basic pathways (particularly metabolic pathways) at a general, conserved annotation level. The simplicity and ethical advantages of the in ovo model make it particularly valuable as a preliminary screening tool prior to more comprehensive studies using established animal preclinical models.

## Glossary

PGC: Primordial germ cell
QTLs: Quantitative trait loci
IOF: In ovo feeding
HMGCR: 3-Hydroxy-3-Methylglutaryl-CoA Reductase
CYP7A1: Cytochrome P450 Family 7 Subfamily A Member 1
miRNAs: MicroRNAs
lncRNAs: Long Non-Coding RNAs
6mA: *N6*-methyladenine
5mC: 5-Methylcytosine
H3K9me2: Histone 3 Lysine 9 Dimethylation
H3K9me3: Histone 3 Lysine 9 Trimethylation
Zeb1: Zinc Finger E-Box Binding Homeobox 1
BMP4: Bone Morphogenetic Protein 4
GPI: Glucose Phosphate Isomerase
C1EIP: Chromosome 1 Expression in PGCs
STAT3: Signal Transducer and Activator of Transcription 3
ENO1: Enolase 1
TGF-β: Transforming Growth Factor Beta
piRNA: PIWI-Interacting RNA
CIWI: PIWI-Like Protein 1
CILI: PIWI-Like Protein 2
HH: Hamburger-Hamilton Stages
SSEA-1: Stage-Specific Embryonic Antigen-1
SSEA-4: Stage-Specific Embryonic Antigen-4
SSEA-3: Stage-Specific Embryonic Antigen-3
POUV: POU Class 5 Homeobox 1 (also known as OCT4 in mammals)
SOX2: SRY-Box Transcription Factor 2
NANOG: NANOG Homeobox (Pluripotency-Associated Transcription Factor)
DAZL: Deleted in Azoospermia-Like
CVH: Chicken Vasa Homolog
FGF2: Fibroblast Growth Factor 2
IGF-1: Insulin-like Growth Factor 1
E: Embryonic day
F1: First Filial Generation
F2: Second Filial Generation
F3: Third Filial Generation
